# Health-promoting behavior and its determinants towards non-communicable diseases among adult residents of the Gedeo zone, South Ethiopia: the application of the health belief model

**DOI:** 10.3389/fpubh.2024.1453281

**Published:** 2024-09-04

**Authors:** Habtamu Endashaw Hareru, Tizalegn Tesfaye Mamo, Mesfin Abebe, Berhanu Gidisa Debela

**Affiliations:** ^1^School of Public Health, College of Health Sciences and Medicine, Dilla University, Dilla, Ethiopia; ^2^Department of Midwifery, College of Health Sciences and Medicine, Dilla University, Dilla, Ethiopia

**Keywords:** Health-promoting Lifestyle Profile II, non-communicable diseases, health belief models, determinants, Ethiopia, health-promoting behavior

## Abstract

**Background:**

Non-communicable diseases are becoming a challenge for the health care system in Ethiopia, which has suffered a double burden from infectious and rapidly increasing non-communicable diseases. However, there is little information on health-promoting behavior in the study settings. Thus, the purpose of this study was to determine health-promoting behaviors and its associated factors among adult's residents of Gedeo zone.

**Methods:**

A cross-sectional study was conducted among 705 adult residents of Gedeo zone, south Ethiopia, selected using a multi-stage sampling technique. Interviews administered through a structured questionnaire were used to collect the data. The data were entered using Kobo Collect and analyzed using Stata version 17. The baseline characteristics of the participants were summarized using descriptive statistics. The independent sample *t*-test and one-way ANOVA were used to compare two groups and more than two groups, respectively. Stepwise multiple linear regression analysis was used to identify the potential determinants of health-promoting behavior and its components. Statistically significant factors were declared at *p*-value of less than or equal to 0.05.

**Results:**

The overall means score for health-promoting behavior was 73.88 ± 16.79. Physical activity and spiritual growth had the lowest and highest mean scores, respectively. The variables: gender, marital status, education, family history of NCDs, health insurance status, perceived health status, knowledge of NCD risk factors, risk perception of NCDs, expected outcome, cues to action, and self-efficacy showed a statistically significant difference in overall health-promoting behavior. The total health-promoting behavior score was associated with age, gender, perceived health status, marital status, family history of NCDs, health insurance, knowledge of NCD risk factors, perceived threat, expected outcome, self-efficacy, and cues to action.

**Conclusion and recommendations:**

In the study, the mean score of health-promoting behaviors was low. Socio-demographic and economic variables, family history of NCD, perceived health status, knowledge of NCD risk factors, perceived threat, expected outcome, self-efficacy, and cues to action affect health-promoting behaviors. Therefore, the study suggests establishing health promotion programs to increase residents' awareness of health-promoting lifestyles, empower them to adopt healthy lifestyles, and improve health outcomes by increasing self-efficacy, providing education, and creating supportive environments.

## Introduction

Non-communicable diseases (NCDs) are medical conditions that cannot be spread from person to person and are one of the major global health challenges (1). These diseases include cardiovascular disease, cancer, diabetes, respiratory diseases (i.e., asthma and chronic obstructive pulmonary disease), arthritis, chronic kidney diseases, and mental health problems (2, 3). Among the NCDs, cardiovascular diseases, cancers, diabetes, and chronic lung diseases account for 80% of the global disease burden. These four groups of diseases account for 74% of all deaths globally and place increasing demands on health systems. Of all NCD deaths, 77% are in low- and middle-income countries, and these four groups of diseases account for over 80% of all premature NCD deaths (4). According to the World Health Organization's (WHO) projection for 2030, the disability-adjusted life years (DALYs) and deaths attributed to NCDs will be 52 million, which is three times greater than those of communicable diseases, rising from 38 million in 2012. Eighty percent of all deaths and over ninety percent of premature mortality in low- and middle-income countries, like Ethiopia, are attributable to NCDs (3). The WHO 2018 country profile report states that non-communicable diseases are responsible for roughly 39% of fatalities in Ethiopia (5).

The incidence of NCDs is rising quickly, placing a significant burden on society and the health care system in low-income nations (6). NCDs are very common and expensive, although the majority of them can be avoided in easy and inexpensive ways. One important way to promote health and prevent disease is through health promotion (7). Health-promoting behaviors are self-initiated actions that enhance wellness, self-actualization, and fulfillment (8). Encouraging these behaviors can reduce the risk of chronic diseases, improve wellbeing, prevent disease, and decrease healthcare costs, leading to improved quality of life, reduced asthma symptoms, and improved cardiovascular outcomes (9, 10). Among the multi-factorial causes of NCDs, behavioral factors like tobacco use, consumption of unhealthy diets, physical inactivity, overdosing on alcohol, and air pollution are identified as major shared risk factors for NCDs (11, 12).

According to WHO projections, by 2025, there will be 1.27 billion tobacco users worldwide, and over 14% of those over the age of 18 are expected to be obese (13, 14). A nationwide study conducted by the Ethiopian Public Health Institute shows that about 4.2% of participants were current smokers, of which 10% and 13% were second-hand smokers at home and at work, respectively. This study also shows that about 41% of study participants consumed alcohol within the past 30 days (15). The 2030 United Nations Sustainable Development Goal (SDG) target 3.4 aims to reduce premature mortality from NCDs by one-third (16). In addition, by 2025, the Ethiopian government is working to reduce the overall premature mortality from NCDs by one-fourth (6). To achieve the goals of health promotion, the strategies focus on removing the above-mentioned behavioral risk factors. There is ample evidence that links some lifestyle choices, such as a healthy diet, low tobacco use, limited alcohol consumption, and physical activity, to non-communicable diseases (11, 17). The World Health Organization (WHO) projects that at least 80% of heart disease, stroke, and type 2 diabetes mellitus can be avoided by removing the key risk factors of non-communicable illnesses (18). In addition, a recent study on type 2 diabetes mellitus (T2DM) shows a normalization of blood glucose levels in patients without therapeutic intervention through significant lifestyle modification, which is the best indicator of health promotion effectiveness (19).

Although adopting lifestyle changes is the ideal strategy for health promotion and preventing non-communicable diseases, health promotion behavior is not widely studied in Sub-Saharan Africa (i.e., Ethiopia), which faces a double burden of infectious and rapidly rising non-communicable diseases. The health belief model (HBM) is a best-fit framework to explain and interpret health-related behaviors. According to the model, individuals' likelihood of taking action is influenced by their perceptions of risk, their perceptions of disease severity, the benefits of interventions and barriers to interventions, cues to action, and their self-efficacy through a reasoning process. Hence, an individual is more likely to take action for healthy behavior in NCDs if he has a high perceived threat (perceived susceptibility ± perceived severity), if his perceived benefits of the intervention outweigh his perceived barriers, if he is capable of adopting the intervention (self-efficacy), and if he has some motivators or reminders to act (cues to action) (20). Therefore, assuming the fitness of the model to evaluate healthy behavior practices, we used HBM to assess health-promoting behavior and its determinants toward NCDs among adult residents of the Gedeo zone, south Ethiopia.

## Materials and methods

### Study area, period, and design

This study was conducted among residents of the Gedeo zone. The Gedeo zone is found in the South Ethiopia region, which is the newly formed 12th regional state in Ethiopia. It extends south as a narrow strip of land along the eastern escarpment of the Ethiopian highlands into the Oromia Region, which borders the Zone on the east, south, and west; the Gedeo Zone shares its northern boundary with the Sidama Region. The total population of the study area is more than 1,226,779. The zone has eight Woreda (Bule, Gedeb, Wonago, Kochire, Dilla Zuriya, Chorso, Reppe, and Yirgachefe) and four administrative towns (Dilla town, Gedeb town, Chelelektu, and Yirgachefe town). Dilla is the zonal town of the Gedeo zone, 359 km from Addis Ababa in the south. In 2014, there were 250,363 households in the zone (source: Gedeo Zone Health Office). The research was carried out from November 01, 2023 to January 01, 2024. A community-based cross-sectional study was conducted to assess health-promoting behavior and its associated factors among adult residents of the Gedeo zone, south Ethiopia.

### Population

All residents of the Gedeo zone were the source population, whereas the study population consisted of individuals from households in selected kebeles that were available during the data collection period. Members of the household in the chosen kebeles who are ≥ 18 years old and less than 65 and have resided for more than 6 months in the study area were included in the study. However, individuals with severe mental illnesses or those whose speech impediments prevented them from answering the questions were excluded.

### Sample size determination, sampling techniques, and procedures

For estimating the sample size, a single mean equation was used $\left(n=\frac{{\left(Z_{\alpha /2}\right)}^{2*}\sigma^{2}}{d^{2}}\right)$ by considering 97% CI (Z_α/2_ = ± 2.17), a margin of error (d) of 3%, overall mean score of health promotion lifestyle Profile II (HPLP-II) (mean ± standard deviation (σ) (126.67 ± 21.29) was obtained from a previous similar study conducted among rural adults in Myanmar (36), with the consideration of the design effect of three (3) and a 10% non-response rate. Finally, the optimum sample size that was used for the current study was 782. A multi-stage sampling technique was employed to choose the study participants. In the first phase, a lottery method (simple random sampling technique) was used to randomly select two Woreda (Wonago and Bule) and one administrative town (Dilla town) out of a total of eight Woreda and four administrative towns, respectively. Nine kebeles (three from each Woreda and three from the administrative town) were chosen at random in the second phase. In the third phase, the number of households in each of the chosen kebeles was documented, and the total sample size (782) was proportionally allocated to each kebele. Then, using a systematic sampling technique (every N/n), the households in each kebele were included in the study. Finally, for households with more than one eligible participant, the interview was done by selecting a single participant using the lottery method, although in the event of a household with no eligible participants, the immediate next household was used. At least two visits were made in cases where eligible respondents were not available at the time of data collection.

### Variables

#### Dependent variable

Health-promoting behavior.

##### Independent variables

Demographic and socioeconomic factors (age (in years), gender, occupation, status of marriage, family size, level of education, and household wealth index). Health insurance status, perceived general health status, family history of NCDs, knowledge about the risk factors of NCDs, and perceived risk perception (perceived threats) (i.e., perceived susceptibility and severity), perceived benefit and perceived barrier (expected outcome of health intervention toward NCDs), cue to action, and self-efficacy toward NCDs.

### Measurements

#### Health-promoting behavior (HPB)

The second version Health-Promoting Lifestyle Profile II (HPLP II) was applied to assess the health-promoting behavior among residents of the Gedeo zone, which was adapted from previous studies (22, 23). The questionnaire consists of 52 items that assess the HPB in six components (i.e., health responsibility, spiritual growth, interpersonal relationship, nutrition, physical activity, and stress management). The three components—health responsibility, spiritual growth, interpersonal relationships, and nutrition—have nine items, respectively. While the last two components—physical activity and stress management—had eight questions each, each component was assessed using a self-reported questionnaire with a four-point Likert scale [i.e., never (1), sometimes (2), often (3), and regularly (4)]. The HPLP II total score ranges from 52 to 208. Except for physical activity and stress management, which have respective ranges of 8 to 32, the total score ranges for all subscales are 9–36. According to Penders, the English version's subscales had reported alpha coefficients ranging from 0.79 to 0.87, the 52-item overall score alpha coefficient of internal consistency was 0.94, and the test-retest reliability was 0.89 (24). Using Cronbach's alpha, the reliability coefficients for the HPLP scale as a whole and its dimensions—health responsibility, spiritual growth, interpersonal relationships, nutrition, physical activity, and stress management—were determined in this study to be 0.917, 0.884, 0.484, 0.613, 0.678, 0.806, and 0.753, respectively.

Finally, to measure overall health-promoting behavior, a mean can be obtained on the whole tool, and the means for each subscale can also be determined separately.

#### Risk perception (perceived threats) toward NCDs

Perceived susceptibility and perceived severity were the two dimensions used to measure the risk perception (perceived threat) of NCDs (25, 26). **Perceived susceptibility** was determined by five items of questions; **perceived severity** was assessed by five items of questions. Both dimensions were assessed using a five-point Likert scale (strongly disagree, disagree, neutral, agree, and strongly agree) (27). A higher value indicates a higher risk perception. We calculated the cumulative risk perception score for each participant for each item which ranged from 10 to 50 (28), which was then treated as a continuous variable for analysis. To quantify perceived threats, a binary outcome variable was then developed based on the mean score; as a result, individuals who scored higher than the mean were believed to have a high-risk perception of NCDs, whereas those who scored lower were seen to have a low-risk perception. **Expected outcome (perceived benefits minus perceived barrier):** both perceived benefits and barriers were measured using seven-item questions that were based on a five-point Likert scale (strongly disagree, disagree, neutral, agree, and highly agree). Independent assessments were made to determine the sum score of perceived benefits and barriers. The sum score of the perceived benefits minus the sum score of the perceived barrier was computed to find the expected outcome. In conclusion, an expected outcome was recorded as positive if perceived benefits outweighed perceived barriers; otherwise, a negative expected outcome was considered.

**Cues to action** were assessed using six-item questions, and responses will be recorded based on a five-point Likert scale (strongly disagree, disagree, neutral, agree, and strongly agree). Individual scores for each item were summed up and treated as continuous variables for analysis. The mean score was then determined. Lastly, individuals who scored above the mean were considered to have high cues to action, whereas individuals who scored below the mean were regarded as having low cues to action (29). **Self-efficacy** was measured by using five items based on a five-point Likert scale (strongly disagree, disagree, neutral, agree, and strongly agree) (30). Individual scores for each item were summed up and treated as continuous variables for analysis. The mean score was then determined. Lastly, individuals who scored above the mean were considered to have high self-efficacy, whereas individuals who scored below the mean were regarded as having low self-efficacy (29). **Knowledge about NCD risk factors:** was assessed by asking participants 10 items of “Yes” or “No” knowledge questions about their knowledge of NCD risk factors (31). The total score (range 0–10) was then determined and categorized based on the mean value. Individuals who correctly answered less than the mean of the knowledge item questions were categorized as having poor knowledge, while those who correctly answered more than the mean of the questions were categorized as having poor knowledge about NCD risk factors.

**Perceived health status:** An individual's assessment of their general health is measured by their perceived health status. A five-point Likert scale was used to rate the participants' health: very good, good, medium, poor, and very poor. When respondents indicated good or very good, their perceived health state was viewed as good; when they indicated poor or very poor, it was regarded as poor; and medium when they considered their health status as “medium”.

**Household wealth index:** We used Principal Component Analysis (PCA) to reduce the dimensionality of large data sets and determine the wealth index of the household. Various domestic assets, such as durable assets, goods assets, domestic animals, and other household materials, were employed as PCA variables. Every asset variable was converted into a binary code. Since they added nothing to the analysis, asset variables with zero standard deviations were removed from the PCA. Using the first PCA component, the wealth quantiles were generated (32). An aggregated score was determined for every surveyed household based on the PCA weights for each asset variable. These scores were classified into quantiles, with quantile 1 (Q1) denoting the 20% of households in the sample who are the poorest and quantile 5 (Q5) indicating the 20% of the richest households. The research participants were categorized into quantiles (the poorest, poorer, middle, richer, and richest). In this study, we have reclassified the household wealth index into three categories: poor, middle, and rich. The rich and richest quantiles make up the rich group, while the poor group is made up of the poorest and poor wealth quantiles.

### Definition of terms

**Non-communicable disease** is a chronic illness with a prolonged duration that is not spread from one person to another (5).

**Common NCDs:** diabetes, cancer, chronic respiratory illnesses such as chronic obstructive pulmonary disease and asthma, cardiovascular disorders (including heart attacks and strokes), and cancer are the four main categories of NCDs (5).

**Perceived susceptibility:** personal assessments of the probability that a person will currently or in the future develop NCDs (25).

**Perceived severity:** personal assessments of how serious NCDs are and how they affect various elements of a person's life (25).

**Perceived benefits:** people's beliefs about whether or not a health behavior will help them manage a health risk (33).

**Perceived barriers:** people's beliefs about whether the costs or negative aspects of adopting a health behavior will prevent them from doing so (33).

**Cues to action:** These motivating factors initiate the decision to follow suggestions on promoting one's health (34).

**Self-efficacy**: personal ability to organize and carry out a specific health behavior; self-assurance that the health behavior will be carried out correctly (30).

### Data collection techniques, procedures, and quality control

The data was collected through the use of an interview-administered questionnaire that was developed by adapting various prior studies on health promotion behavior and its associated factors (8, 22–24, 27, 34, 35). A questionnaire designed and distributed using the Kobo Toolbox was used for data collection. The tools comprise data about participants' socio-demographic and economic characteristics, family history of chronic disease, health insurance, perceived health status of the respondent, social support, risk perception toward NCDs, perceived self-efficacy, knowledge about the risk factors of NCDs, and HPLP-II questionnaires. Four trained data collectors and two supervisors collected the data who are proficient in both Amharic and Gedeo languages and are from the health science field. The participants were made aware of the study's objectives, any possible risks and advantages of participation, the study's confidentiality, and their right to decline participation or withdraw from the study at any time before the actual data collection begins. Each participant provided their verbal or written informed consent before the data were gathered. Participants in the study were interviewed in their homes, and data collectors visited every household in each of the chosen kebeles until the required sample size was attained. The period of data collection was from November 01, 2023, to January 01, 2024. The questionnaire was first written in English, then translated into the local language (Amharic or Gedeuffa), and then, in order to achieve consistency, it was translated back into English. Before the actual data collection period, the data collectors received two days of training on the purpose of the study, questionnaire administration procedures, and how to protect study participants' privacy and confidentiality. The structured questionnaire, which was adapted from various literatures, was pre-tested on 5% of the total sample size in Kebeles within the same town that was excluded from the actual study. The questionnaire was modified in accordance with the results of the pre-test. At the end of each data collection session, the data collectors were observed, and the collected data were checked for completeness.

### Statistical data processing and analysis

After being collected with a Kobo Toolbox, the data were exported to an Excel spreadsheet, checked for completeness, consistency, and missing values, and then coded before being loaded into STATA version 17 for analysis. To summarize categorical data, frequencies and percentages were used, while means with standard deviation (SD) or median with interquartile range (IQR) were computed for continuous data. The Shapiro-Wilk test was used to assess the normality of the data, and the non-significant (*p* > 0.05) result indicates normally distributed data. The homogeneity of variance was tested before running a one-way analysis of variance (ANOVA), in which the non-significant result (*p* > 0.05) was used to declare homogeneity of variance, supporting the null hypothesis of equal variance between the two groups. Moreover, we have conducted a Welch F test for data that violates the homogeneity of variance (*p* < 0.05) during one-way ANOVA analysis.

To compare the overall mean difference of HPB and its components among different predictors, the independent sample *t*-test and one-way ANOVA were computed. To confirm the significant differences between each specific group of variables, Tukey's honestly significant difference (HSD) or Scheffe *post-hoc* test was determined for the data that met the assumption of homogeneity of variance, while the Games Howell *post-hoc* test was used for the data that didn't meet the assumption of homogeneity of variance. *Post-hoc* tests were computed for only variables that had an overall statistically significant difference in group means (a statistically significant result in a one-way ANOVA).

Using Akaike's information criterion (AIC), the model's goodness of fit was checked. A Q-Q plot was employed to evaluate the data's normality; no transformation was required; outliers were found to be less significant; and the data was kept in the final model. To test for co-linearity among predictor variables, the variance inflation factor, or VIF, was employed; a threshold value of < 10 shows no multicollinearity; in this study, the VIF for all variables was less than three, indicating the absence of multicollinearity.

Stepwise multiple linear regression analysis was carried out for the overall HPB score and each of the HPB subscales to assess the predictive ability of various combinations of predictor variables. Each variable was simultaneously added to a stepwise regression analysis, where the significance of each new variable was evaluated until the best-fitting model was found. Finally, variables with *p* ≤ 0.05 were considered statistically significant predictors of health-promoting behavior and its subscales. Parameter estimates (β), *p*-values, and the total amount of variance explained by the models in R squared. are displayed as the linear regression results.

### Ethical approval and consent to participate

The ethical approval letter was obtained from the Institutional Review Board (IRB) of Dilla University, College of Health Sciences and Medicine, with the ethical protocol number duchm/IRB/044/2023. This ethical approval letter was provided to responsible administrative offices and other concerned bodies to grant official permission. Each participant was requested to give verbal informed consent after being told about the objectives of the study. Participants were notified, and they were entirely free to accept or refuse this consent at their choice. All phases of the research endeavor maintained the confidentiality and anonymity of the data obtained.

## Result

### Socio-demographic and economic characteristics of the participants

Out of the total 782 sample sizes, 705 participants were chosen for this study, resulting in a 90.15% response rate. Participants were at least 18 years of age. We computed the median age as the data was asymmetrically distributed (skewed to the right), and the participants' median (IQR) age was 37 (28–45) years. Of the participants, 433 (61.42%) were women. The family size ranged from 1 to 10, with a mean (SD) of 4.38±1.66. Of the total participants, 533 (71.35%) were married, and above one-third (35.89%) had completed secondary school. Out of the total 705 participants, around 32.77% were housewives, and 33.05% belonged to the rich category according to the household wealth index.

Furthermore, 39.35%, 61.13%, and 71.21% of the participants claimed to have a family history of NCDs, health insurance, and good general health status, respectively (Table 1).

**Table 1:** Socio-demographic and economic characteristics of residents of Gedeo zone, South Ethiopia region, 2023 (*n* = 705).

**Variables**	**Frequency**	**Percentage (%)**
**Gender**		
Female	433	61.42
Male	272	38.58
**Age (median)** = **37 (IQR: 28–45) years**		
**Family size (mean** = **4.38 and standard deviation** = **1.66)**		
**Marital status**		
Single	155	21.99
Married	503	71.35
Divorced	47	6.67
**Educational status**		
No formal education	48	6.80
Grades 1–8 (primary)	96	13.62
Grades 9–12 (secondary)	253	35.89
College and above	308	43.69
**Occupational status**		
Student	90	12.77
Government employed	208	29.50
Merchant	125	17.73
Housewife	231	32.77
Others^*^	51	7.23
**Households' wealth index**		
Poor	223	31.63
Middle	249	35.32
Rich	233	33.05
**Family History of NCDs**		
No	428	60.71
Yes	277	39.29
**Health insurance**		
No	274	38.87
Yes	431	61.13
**Perceived general Health status**		
Poor	82	11.63
Moderate	121	17.16
Good	502	71.21

^*^Farmer, daily laborer, and pension.

### Respondents' knowledge and perceptions toward NCDs

Individual risk perceptions, one of the six HBM components, relate to an individual's perception of the illness's perceived severity and risk of contracting it (perceived susceptibility), and in this study, over half (54.33%) of the participants had a high-risk perception toward non-communicable diseases. Almost three-fourths of the participants had positive expected outcomes from their health actions. More than half (55.74%) of the participants had high cues to action and self-efficacy toward health behavior, respectively. Moreover, more than three-fourths (76.32%) of the participants had good knowledge about NCD risk factors (Table 2).

**Table 2:** The perception of NCDs and knowledge about NCD risk factors among residents of the Gedeo zone, South Ethiopia region, 2023 (n = 705).

**Variables**	**Frequency**	**Percentage (%)**
**Risk perception of NCDs**		
Low perception	322	45.67
High perception	383	54.33
**Expected outcome**		
Negative	183	26.00
Positive	522	74.00
**Cues to action**		
Low	323	45.80
High	382	54.20
**Self-efficacy**		
Low	312	44.26
High	393	55.74
**Knowledge about NCDs**		
Poor	167	23.69
Good	538	76.31

### Health-promoting behavior of the participants toward NCDs

In this study, the overall mean score for health-promoting behavior was 73.88 ± 16.79. The mean scores of the six HPLP subscales were higher for the spiritual growth subscale (16.8 ± 2.9), while the physical activity subscale had the lowest score (6.16 ± 4.37) (Table 3).

**Table 3:** The overall score of health-promoting behavior and its subscales among adult residents of Gedeo Zone, South Ethiopia region, 2023 (n = 705).

**Health-promoting behavior scales**	**Possible range score**	**Mean (±SD)**	**Range**
Health responsibility	9–36	11.6 ± 5.5	2–22
Spiritual growth	9–36	16.8 ± 2.9	9–23
Interpersonal relationship	9–36	13.5 ± 2.8	7–23
Nutrition	9–36	13.6 ± 3.4	5–25
Physical activity	8–32	6.16 ± 4.4	0–22
Stress management	8–32	12.1 ± 2.9	4–19
Overall score of HPLP II	52–208	73.88 ± 16.8	38–115

### Differences in health-promoting behaviors and its subscales based on participants' baseline characteristics

In regards to differences in health–promoting behavior based on participant socioeconomic demographic attributes the results of the independent sample t–test and ANOVA indicates, gender (t = −6.89, *p* < 0.01), occupational status (F = 41.72, *p* < 0.01), educational status (F = 35.52, *p* < 0.01), marital status (F = 14.5, *p* < 0.01), family history of NCDs (t = 2.048, *p* = 0.041), health insurance status (t = −5.25, *p* < 0.01), perceived health status (F = 93.83, *p* < 0.01), knowledge about NCDs risk factors (t = – 8.99, *p* < 0.01), risk perception of NCDs (t =-5.85, *p* < 0.01), expected outcome (t = −7.98, *p* < 0.01), cues to action (t =-16.75, *p* < 0.01), and self–efficacy (t =-20.90, *p* < 0.01) showed a statistically significant difference with overall health-promoting behavior.

To compare the specific mean difference between each group, the Games-Howell *post-hoc* test was used because the assumption of homogeneity of variance was not met. The results indicated that, in terms of occupational status, students (mean score of 89.49 ± 0.42) had significantly higher overall HPB scores than government employees (76.52 ± 15.00), merchants (73.68 ± 12.05), housewives (68.21 ± 15.84), and other occupations (61.82 ± 14.29). According to the results of the Games-Howell *post-hoc* test, participants with college and above (mean score = 77.95 ± 17.75) had a statistically significant higher overall HPB score than participants with no formal education (68.10 ± 18.10) and primary education (59.83 ± 10.44). According to the Games-Howell *post-hoc* test, participants with single marital status (mean = 80.00 ± 21.30) had a statistically significant higher HPB than those with married status (71.89 ± 15.17). The Games-Howell *post-hoc* test showed that participants with good perceived health status had a significantly higher overall HPB score than those with poor (60.95 ± 11.91) and moderate perceived health status (62.34 ± 10.62).

The study found that gender, occupational status, educational status, marital status, perceived health status, family history of non-communicable diseases (NCDs), and perceived outcome all significantly influenced health-promoting behaviors (HPB) subscales. Students had higher health responsibility, nutrition, and physical activity compared to other occupations, while those with secondary education had higher health responsibility and spiritual growth. Marital status also played a role in HPB subscales, with single participants having higher physical activity scores and spiritual growth. Household wealth index showed no significant difference in overall HPB scores, but it showed significant differences in spiritual growth, interpersonal relationships, and nutrition. Perceived health status also showed significant differences across HPB subscales. Family history of NCDs also showed significant differences in spiritual growth and stress management subscales. Risk perception for NCDs showed significant differences in stress management, physical activity, nutrition, spiritual growth, and health responsibility. Cues to action and self-efficacy also showed significant differences across all HPB subscales (Table 4).

**Table 4:** Relationship between the overall score of the health-promoting behavior and its subscales with the baseline characteristics of adult residents of the Gedeo zone, South Ethiopia region, 2023 (n = 705).

		**Overall score**	**HR**	**SG**	**IR**	**Nu**	**PA**	**SM**
**Variables**	**Category**	**Mean (SD)**						
Gender	Female	70.54 (16.1)	11.69 (5.3)	16.35 (3.1)	13.30 (3.0)	12.9 (3.3)	4.49 (2.8)	12.02 (2.9)
	Male	79.21 (16.6)	11.87 (5.5)	17.51 (2.5)	13.97 (2.3)	14.7 (3.4)	8.81(5.1)	12.44 (2.9)
	Df *t*(p)	703 −6.89 (< 0.01)	703 −0.41 (0.68)	703 −5.24 (< 0.01)	703 −3.12 (< 0.01)	703 −7.26 (< 0.01)	703 −14.53 (< 0.01)	703 −1.88 (< 0.01)
Occupation	Student	89.49 (17.7)	15.43 (4.9)	18.41(2.4)	14.17 (1.8)	15.62 (3.4)	12.22 (5.7)	13.77 (2.9)
	Government employee	76.52 (15.0)	11.49 (5.0)	17.45 (2.9)	14.05 (3.4)	14.19 (3.4)	6.85 (3.5)	12.70 (2.7)
	Merchant	73.68 (12.1)	11.62 (5.2)	16.91(2.3)	13.21(2.7)	14.14 (3.0)	6.06 (3.5)	11.98 (2.3)
	House wife	68.21(15.8)	11.07 (5.5)	15.86 (3.0)	3.32 (2.5)	12.71 (3.2)	3.77 (2.4)	11.71(3.0)
	Other^*^	61.82 (14.3)	9.94 (5.0)	15.33 (2.8)	12.53 (2.3)	10.65 (2.8)	3.75 (1.5)	9.96 (3.3)
	Df F(p)	700 41.72 (< 0.01) a > b, c, d, e	700 13.96 (< 0.01) a > b, c, d, e	700 20.96 (< 0.01) a > b, c, d, e	700 5.48 (< 0.01) a > e	700 26.69 (< 0.01) a > b, c, d, e	700 103.86 (< 0.01) a > b, c, d, e	700 18.77 (< 0.01) a > c, d, e
Educational status	No formal edu.	68.10 (18.1)	10.44 (5.9)	15.96 (3.1)	13.13 (3.1)	12.38 (3.7)	4.73 (3.6)	11.73 (2.9)
	Primary	59.83 (10.4)	8.66 (3.8)	14.00 (2.4)	12.11(1.6)	11.54 (2.3)	3.21 (2.2)	10.69 (2.3)
	Secondary	75.37 (14.7)	13.02 (5.4)	17.21(2.5)	13.59 (2.6)	13.96 (3.2)	5.33 (3.7)	12.47 (2.8)
	College and above	77.95 (17.8)	11.92 (5.3)	17.47 (2.9)	14.07 (3.0)	14.19 (3.6)	8.00 (4.7)	12.49 (3.1)
	Df F(p)	701 35.52 (< 0.01) i > f, g	701 17.37 (< 0.01) h > f, g i > g	701 45.25 (< 0.01) i > f, g h > f, g	701 12.98 (< 0.01) h >g i > g	701 18.62 (< 0.01) h >f, g i > f, g	701 44.32 (< 0.01) h >f, g i > f, g, h	701 11.06 (< 0.01) h > g i > g
Marital status	Single	80.00 (21.3)	12.25 (5.9)	17.32 (3.3)	13.98 (2.9)	14.06 (3.9)	9.97 (5.8)	12.54 (3.7)
	Married	71.89 (15.2)	11.61 (5.3)	16.61(2.8)	13.41 (2.8)	13.46 (3.2)	5.03 (3.2)	12.02 (2.7)
	Divorced	75.13 (10.8)	11.85 (4.2)	17.11 (2.3)	13.89 (2.0)	13.89 (3.8)	5.79 (2.6)	12.74 (2.4)
	Df F(p)	702 14.50 (< 0.01) j > k	702 0.85 (< 0.43)	702 3.81 (< 0.05) j > k	702 2.85 (0.06)	702 1.99 (0.14)	702 96.60 (< 0.01) j > k >l	702 2.77 (0.06)
Households' wealth index	Poor	75.23 (13.2)	11.36 (4.7)	17.81 (1.9)	14.22 (2.8)	13.99 (3.2)	6.08 (4.1)	12.02 (2.6)
	Medium	71.99 (18.7)	12.27 (5.7)	16.02 (3.1)	13.11 (2.6)	12.57 (3.4)	6.09 (4.4)	12.10 (3.4)
	Rich	74.63 (17.59)	11.61 (5.65)	16.67 (3.26)	13.42 (2.91)	14.39 (3.48)	6.33 (4.66)	12.44 (2.72)
	Df F(p)	702 2.54 (0.08)	702 1.84(0.16)	702 24.0 (< 0.01) m > n, o	702 9.92 (< 0.01) m > n, o	702 19.53 (< 0.01) m > n; o > n	702 0.24(0.79)	703 1.32(0.27)
Family Hx of NCDs	No	74.89 (15.6)	11.97 (5.4)	17.23 (2.5)	13.64 (2.6)	13.75 (3.4)	6.07 (3.9)	12.42 (2.9)
	Yes	72.25 (18.4)	11.43 (5.3)	16.24 (3.4)	13.32 (3.0)	13.39 (3.6)	6.31 (5.0)	11.81 (2.9)
	Df t(p)	702 2.05 (< 0.05)	702 1.31(0.19)	702 4.91 (< 0.01)	702 1.04(0.29)	702 1.35(0.18)	702 −0.72(0.48)	702 2.71 (< 0.05)
Health insurance	No	69.79 (16.2)	11.56 (5.4)	15.83 (3.1)	13.19 (2.8)	13.19 (3.3)	4.92 (3.3)	11.39 (3.2)
	Yes	74.48 (16.7)	11.89 (5.4)	17.42 (2.6)	13.80 (2.8)	13.89 (3.8)	6.95 (4.8)	12.69 (2.7)
	Df t(p)	703 −5.25 (< 0.01)	703 −0.82(0.42)	703 −7.32 (< 0.01)	703 −2.87 (< 0.05)	703 −2.62 (< 0.05)	703 −6.20 (< 0.01)	703 −5.87 (< 0.01)
Perceived Health status	Poor	60.95 (11.9)	7.50 (2.8)	15.12 (3.3)	12.98 (2.1)	11.77 (3.5)	3.89 (3.1)	10.09 (1.8)
	Moderate	62.34 (10.6)	6.85 (2.7)	15.88 (2.8)	12.74 (3.1)	12.07 (2.7)	4.29 (2.7)	10.72 (2.1)
	Good	78.78 (16.2)	13.65 (5.0)	17.30 (2.7)	13.86 (2.8)	14.30 (3.4)	6.99 (4.6)	12.88 (3.0)
	Df F(p)	702 93.83 (< 0.01) r > p, q	702 152.41 (< 0.01) r > p, q	702 29.12 (< 0.01) r > p, q	702 10.11 (< 0.01) r > p, q	702 37.16 (< 0.01) r > p, q	702 34.00 (< 0.01) r > p, q	702 58.32 (< 0.01) r > p, q
Knowledge about NCDs	Poor	64.21 (14.7)	9.07 (4.8)	15.14 (3.4)	13.32 (3.2)	11.71 (3.2)	4.89 (3.5)	10.32 (2.6)
	Good	76.89 (16.3)	12.60 (5.3)	17.31 (2.5)	13.64 (2.7)	14.21 (3.3)	6.55 (4.5)	12.76 (2.8)
	Df t(p)	703 −8.99 (< 0.01)	703 −7.69 (< 0.01)	703 −8.85 (< 0.01)	703 −1.28(0.19)	703 −8.61 (< 0.01)	703 −4.33 (< 0.01)	703 – 10.05 (< 0.01)
Risk perception of NCDs	Low	69.94 (15.9)	11.47 (4.9)	15.78 (2.9)	13.40 (2.8)	12.99 (3.3)	5.07 (3.9)	11.45 (2.9)
	High	77.20 (16.8)	12.01 (5.8)	17.65 (2.6)	13.70 (2.8)	14.14 (3.5)	7.08 (4.5)	12.80 (2.8)
	Df t(p)	703 −5.85 (< 0.01)	703 −1,31 (0.19)	703 −8.97 (< 0.01)	703 −1.41 (0.16)	703 −4.49 (< 0.01)	703 −623 (< 0.01)	703 −6.24 (< 0.01)
Expected outcome	Negative	65.72 (15.5)	9.85 (5.3)	15.47 (3.0)	13.31 (2.5)	12.56 (3.4)	4.08 (2.3)	10.74 (3.0)
	Positive	76.75 (16.3)	12.44 (5.3)	17.27 (2.7)	13.65 (2.9)	13.99 (3.4)	6.89 (4.7)	12.69 (2.8)
	Df t(p)	703 −7.98 (< 0.01)	703 −5.72 (< 0.01)	703 −7.44 (< 0.01)	703 −1.40 (0.16)	703 −4.91 (< 0.01)	703 −7.82 (< 0.01)	703 −8.16 (< 0.01)
Cues to action	Low	64.14 (14.4)	9.15 (5.1)	15.88 (3.0)	12.78 (2.3)	11.71 (3.2)	4.06 (2.4)	10.87 (2.7)
	High	82.13 (14.1)	13.98 (4.6)	17.58 (2.6)	14.23 (3.0)	15.24 (2.8)	7.94 (4.9)	13.29 (2.7)
	Df t(p)	703 −16.75 (< 0.01)	703 −13.22 (< 0.01)	703 −8.03 (< 0.01)	703 −7.07 (< 0.01)	703 −15.78 (< 0.01)	703 −13.07 (< 0.01)	703 −11.99 (< 0.01)
Self– efficacy	High	62.22 (12.9)	7.38 (3.7)	15.44 (3.0)	12.79 (2.9)	11.63 (2.9)	5.00 (3.3)	10.26 (2.4)
	Low	83.14 (13.4)	15.24 (3.7)	17.88 (2.3)	14.17 (2.5)	15.19 (3.0)	7.08 (4.9)	13.71 (2.4)
	Df t(p)	703 −20.90 (< 0.01)	703 −27.93 (< 0.01)	703 −12.15 (< 0.01)	703 −6.69 (< 0.01)	703 −15.86 (< 0.01)	703 −6.42 (< 0.01)	703 −19.00 (< 0.01)

HX, history; ^*^statistically significant at p < 0.01; ^*^statistically significant at p < 0.05; SD, standard deviation; HR, health responsibility; SG, spiritual growth; IR, interpersonal relation; Nu, nutrition; PA, physical activity; SM, stress management; a, students; b, government employee; c, merchants; d, housewife; e, other^*^ (unemployed, farmers, pension); f, no formal education; g, primary; h, secondary; i, college and above; j, single; k, married; l, divorced; m, poor; n, medium; o, rich; p, poor; q, moderate; r, good.

### Factors associated with health-promoting behaviors

A separate regression analysis was used to examine the predictive power of each predictor on the overall HPB score and its subscales. According to the results of the stepwise regression analysis, self-efficacy was the most potent factor impacting behaviors that promote health (β = 14.67, *p* < 0.01). The participants' age (β = −0.39, *p* < 0.01) was the second most significant predictor, followed by gender of the participants (β = 7.29, *p* < 0.01), cues to action (β = 5.16, *p* < 0.01), knowledge of the risk factors for NCDs (β = 5.85, *p* < 0.01), and the least significant predictor was risk perception of NCDs (β = 2.34, *p* < 0.05). These variables have 66.0% power to predict (*p* < 0.01, R^2^ = 0.660, Adj. R^2^ = 0.652) the health–promoting behaviors.

Based on the health–promoting behavior subdomains, the strongest predictor affecting health responsibility was self–efficacy (β = 5.77, *p* < 0.01).

The predictive power of those variables on the health responsibility subscale was 67.4% (*p* < 0.01, R^2^ = 0.674, Adj. R^2^ = 0.688).

A substantial number of variables predicted spiritual growth: the highest ranked variables were self–efficacy (β = 2.19, *p* < 0.01), risk perception of NCDs (β = 0.85, *p* < 0.01), and thorough knowledge of NCDs (β = 1.44, *p* < 0.01).

All significant predictors accounted for 51.1% of the variation in spiritual growth (*p* < 0.01, R^2^ = 0.551, adj. R^2^ = 0.501).

The factors that had the greatest impact on the interpersonal relation subscale were self–efficacy (β = 0.93, *p* < 0.01) and cues to action (β = 1.01, *p* < 0.01). Predictors in this model accounted for 18.7% of the variation in interpersonal relations (*p* < 0.01, R^2^ = 0.187, adj. R^2^ = 0.184).

One of the factors influencing the nutrition subscale was self–efficacy (β = 2.43, *p* < 0.01), along with cues to action (β = 1.60, *p* < 0.01). This model showed that 50.4% of the variation in the nutrition subscale was explained by all predictors (*p* < 0.01, R^2^ = 0.504, adj. R^2^ = 0.496).

The most significant factor impacting physical activity was male gender (β = 3.84, *p* < 0.01). All the factors in this model explained 65.8% of the variation in physical activity (*p* < 0.01, adj. R^2^ = 0.652, R^2^ = 0.658).

Moreover, self–efficacy (β = 2.65, *p* < 0.01) was the most potent factor affecting stress management. This model showed that 55.7% of the variation in stress management could be explained by all significant factors (*p* < 0.01, R^2^ = 0.557, adj. R^2^ = 0.549) (Table 5).

**Table 5:** The summary of a stepwise multiple regression analysis of baseline characteristics on the overall score of health-promoting behavior and its subscales among residents of the Gedeo zone, south Ethiopia region (n = 705).

**Variables**	**Overall HPB**	**HR**	**SG**	**IR**	**Nu**	**PA**	**SM**
	**β (SE)**	**β (SE)**	**β (SE)**	**β (SE)**	**β (SE)**	**β (SE)**	**β (SE)**
Constant	71.58 (2.3)	6.27 (0.6)	17.88 (0.5)	16.73 (0.6)	12.53 (0.5)	11.39 (0.6)	9.71 (0.4)
Age	−0.39 (0.1)^**^		−0.05 (0.0)^**^	−0.03 (0.0)^**^	−0.04 (0.0)^**^	−0.15 (0.0)^**^	−0.04 (0.0)^**^
Gender	7.29 (0.9)^**^		0.44 (0.2)^*^		1.40 (0.2)^**^	3.84 (0.3)^**^	
Family size			−0.29(0.1)^**^	−0.33 (0.0)^**^			0.23 (0.1)^**^
Married	3.68 (1.0)^**^	3.24 (0.4)^**^	0.70 (0.2)^**^		0.84 (0.2)^**^		1.25 (0.2)^**^
Divorced		1.94 (0.6)^**^					1.85 (0.4)^**^
Primary school	−6.91 (1.2)^**^	−2.59 (0.4)^**^	−1.66 (0.3)^**^	−0.71 (0.3)^*^	−1.06 (0.3)^**^		−0.62 (0.2)^*^
Collage and above						1.06 (0.3)^**^	
Govt employee	−2.96 (1.0)^*^	– 3.84 (0.5)^**^			−0.61 (0.3)^*^	−2.56 (0.4)^**^	
Merchant	−6.94 (1.2)^**^	−4.61(0.6)^**^		−0.68 (0.3)	−0.96 (0.3)^*^	−2.64 (0.4)^**^	−0.71 (0.2)^*^
Housewife		−3.28 (0.6)^**^	0.82 (0.2)^**^	0.56 (0.2)^*^		−2.59 (0.5)^**^	
Other	−7.41 (1.7)^**^	−3.20 (0.7)^**^			−2.60 (0.4)^**^	−4.24 (0.5)^**^	−1.23 (0.3)^**^
Health insurance		0.61 (0.3)^*^	0.81(0.2)^**^			−0.98(0.3)^**^	0.41(0.2)^*^
Family Hx of NCDS	2.34 (0.8)^*^	0.79 (0.3)^**^				1.31 (0.2)^**^	
Moderate perceived health status		−1.64 (0.5)^**^	−0.56 (0.23)^**^	−0.52 (0.3)^*^			
Good perceived health status	5.39 (1.1)^**^	2.82 (0.4)^**^					
Middle household wealth index	−6.07 (1.1)^**^		−2.31 (0.2)^**^	−1.96 (0.3)^**^	−1.62 (0.2)^**^		
Rich household wealth Index	−3.14(1.0)^*^		−1.77 (0.2)	−1.17(0.3)^**^			
Knowledge on NCDs risk factors	5.85 (1.0)^**^	1.39 (0.3)^**^	1.44 (0.2)^**^		1.64 (0.2)^**^		1.55 (0.2)^**^
Risk perception of NCDs	2.34(0.9)^*^		0.85 (0.2)^**^				0.71 (0.2)^**^
Expected outcome	3.75 (1.1)^**^		0.60 (0.2)	0.81(0.3)^*^	1.09 (0.3)^**^	0.53 (0.3)^*^	
Cues to action	5.16 (1.0)^**^			1.01 (0.2)^**^	1.60(0.2)^**^	2.01(0.3)^**^	0.55 (0.2)^*^
Self-efficacy	14.67 (0.9)^**^	5.77 (0.3)^**^	2.19 (0.2)^**^	0.93 (0.2)^**^	2.43 (0.2)^**^	0.58 (0.2)^*^	2.65 (0.2)^**^
R^2^	0.660	0.674	0.511	0.187	0.504	0.658	0.557
Adjusted -R square	0.652	0.668	0.501	0.184	0.496	0.652	0.549

HR, health responsibility; SG, spiritual growth; IR, interpersonal relations; Nu, nutrition; PA, physical activity; SM, stress management; ^*^p < 0.05, ^**^p < 0.01.

## Discussion

Behaviors that promote health are considered to be among the primary indicators of health; they are the primary causes of many diseases, and these behaviors have a significant impact on both the promotion of health and the prevention of disease. However, there is currently little information available about health-promoting behaviors in Ethiopia. This study attempted to assess the health-promoting behaviors and associated factors among adult residents of the Gedeo Zone, South Ethiopia, within the framework of a health belief model. In this study, the overall mean score of health-promoting behavior was 73.88 ± 16.79. This finding was lower than a study conducted in Myanmar (36), Dena Province (37), Turkey (38, 39), and Iran (40). These differences could be caused by differences in the study population, sample characteristics, sociocultural background, and participant attributes.

Based on the mean score for subscales of health-promoting behavior, spiritual growth was the most commonly practiced in the study area, followed by nutrition, interpersonal relations, stress management, and health responsibility, while physical activity was the least commonly practiced health-promoting behavior, which is consistent with a study conducted in Myanmar (36), Spain (41), and the United States of America (USA) (42). The finding might be because Gedeo society's culture and belief (religious) status have the potential to maintain spiritual growth. These findings highlight the influence of religion on people's values, beliefs, daily routines, and health-promoting behaviors (43). Several factors contribute to the high score on the nutrition subscale, including the Gedeo zone's environmental features and easy access to affordable fruits, vegetables, and dairy products.

The overall health-promoting behavior score in this study, along with each health-promoting behavior sub-scale except the health responsibility sub-scale, demonstrated a statistically significant gender difference. The spiritual growth, nutrition, and physical activity sub-scales, as well as the overall health-promoting behaviors, were positively affected by gender, with males being more likely to practice such behaviors as compared to women. The finding was consistent with previous similar studies (24, 40, 44, 45). This could be because, in comparison to women, men are more likely to meet the requirements for physical exercise (46). The results could be explained by the fact that males frequently participate in traditionally masculine activities like weightlifting and competitive sports, which are influenced by society's expectations and traditional gender norms. Their greater perception of health concerns, such as diabetes, cancer, and heart disease, may potentially have an impact on their health-promoting behaviors. They also have more access to resources and disposable income. Therefore, knowing the causes of gender disparities in health-promoting behaviors might assist in developing tailored interventions and strategies that support male and female health-promoting lifestyles. Healthcare providers and public health experts can strive to promote holistic approaches to health promotion for all individuals and reduce inequities by addressing underlying issues such as societal norms, risk perception, access to resources, and healthcare-seeking behavior.

This study found that, except for the health responsibility subscales, the overall health-promoting behaviors and the subscales showed a negative association with participant age. This means that, for every unit rise in age, the overall health-promoting behaviors and the subscales decreased. This finding was consistent with a study conducted in Tehran (47), Hungary (48), Spain (41), the USA (42), and Iran (49). There could be some reasons for this relationship, including a greater understanding of the value of health, higher levels of physical fitness, and a decline in chronic illnesses among younger age groups. Aging also brings with it a decline in one's everyday physical and mental abilities. Thus, we encourage that adults who practice health-promoting behaviors can benefit from assistance in adjusting to their new circumstances and maintaining a high quality of life, which can dramatically lower the rate of early mortality.

In this study, it was found that there were statistically significant differences between the overall mean health- promoting behavior and its subscales about the following variables: occupational status, level of education, household wealth index, perceived health status, family history of NCDs, marital status, and health insurance status. Primary education levels, middle and high household wealth index, government employee merchants, and others in occupational status were negatively related to the overall health-promoting behavior. This finding was supported by previous studies (36, 49). This result may be explained by lower education levels, which might result in restricted access to health services and information, which can affect the significance of engaging in health-promoting activities. Individuals with middle and wealth index scores are more likely to lead sedentary lives, have poor eating patterns, and have easier access to processed foods and sugary drinks. They might also be more likely to rely on convenience foods, which are frequently high in calories and low in nutritious content, which can lead to unhealthy habits. Government employees and merchants may experience stress at work and unpredictable schedules that make it difficult for them to adopt healthy lifestyles. Additionally, their health-promoting behavior can be affected by a lack of social support. This finding was supported by different studies conducted on health-promoting behavior among adults (36, 41, 42, 50, 51). Therefore, to encourage a culture of health and wellbeing across a range of professions and socioeconomic backgrounds, specific interventions are required.

Health-promoting behavior was positively associated with marital status, with married participants more likely to adopt such behaviors than the corresponding comparison group. These results were consistent with the research that was done in Spain (41). The results could be explained by the fact that marriage creates shared commitments and provides social support, accountability, and encouragement for health-promoting behavior. Health-promoting behaviors are positively affected by perceived health status, and a good perception of one's health can significantly influence the adoption of behaviors that promote health. These findings have been supported by previous research conducted in Spain (41), Korea (21), and Myanmar (36). The finding might be because individuals who assess their health state as good tend to make better decisions regarding self-care and health prevention.

The family history of NCD had a positive association with the subscales measuring physical activity, health responsibility, and overall health-promoting behavior. It may be discovered that an individual's awareness of their personal risk factors and desire to adopt health-promoting behaviors as a preventive measure may be affected by a family history of non-communicable diseases. Additionally, knowing one's genetic predispositions can motivate people to change their lifestyles to reduce the likelihood of developing linked disorders. A study carried out in the Gambia provided support for the findings and a possible explanation of the present study (52).

In this study, the knowledge of NCD risk factors among participants showed statistically significant differences in the subscales measuring stress management, physical activity, nutrition, spiritual growth, and overall health-promoting behaviors, except interpersonal relations. In addition, participants who had good knowledge of the risk factors for NCDs were more likely to undertake health-promoting behavior and its subscales (i.e., health responsibility, spiritual growth, nutrition, and stress management). This finding is consistent with a study conducted among community residents in China (53) and Myanmar (54). The finding may be the result of people being more equipped to make decisions about their health when they are aware of the risk factors for NCDs, which include stress, alcohol use, poor diet, smoking, and inactivity. Understanding the risk factors of NCDs promotes healthy lifestyle choices such as quitting smoking, adopting healthier diets, moderating alcohol intake, engaging in exercise, and handling stress. Along with early intervention and improved treatment of NCD risk factors, it also promotes preventative measures such as routine health screenings and monitoring. Additionally, it promotes lifestyle modification and behavioral changes, which lower the risk factors for NCDs by decreasing alcohol consumption or seeking help to quit drinking.

This study found statistically significant differences between high and low perceived outcomes of health interventions toward NCDs for the mean overall score and all HPB subscales, except the interpersonal relations subscale. Furthermore, participants who reported a high perceived outcome were more likely, compared to those who perceived a low perceived outcome, to engage in the total health-promoting behavior and its subscales (spiritual growth, interpersonal relationships, nutrition, and physical activity). This result was in line with a study carried out in Alexandria (43). The relationship between expected outcomes and overall health-promoting behaviors is crucial to understanding how individuals perceive the advantages of engaging in behaviors that promote their wellbeing (55). By recognizing and promoting the perceived benefits of these behaviors, individuals can be encouraged to adopt and maintain health-promoting behaviors that enhance their overall quality of life and wellbeing.

Regarding perceived self-efficacy, there was statistically significant variation in both the overall health-promoting behavior and its subscales. Furthermore, individuals with high perceived self-efficacy were more likely to engage in health-promoting behaviors, health responsibility, spiritual growth, interpersonal relationships, nutrition, physical activity, and stress management subscales. The results could be explained by the fact that people with high self-efficacy are more likely to participate in behaviors that promote health because they have confidence in their capacity to overcome challenges, are resilient when faced with setbacks, and can establish attainable health goals. This finding was supported by previous studies (21, 56–59). Therefore, the relationship between engaging in health-promoting behaviors and having a high level of self-efficacy highlights the significance of encouraging people to believe they can make changes to improve their health. Healthcare providers and public health experts can enable people to take ownership of their health, adopt healthy behaviors, and achieve better health outcomes by increasing self-efficacy through empowerment, education, and support.

The findings of this study revealed a statistically significant difference in the HPB overall and in each of its subscales between participants with high cues to action and low cues to action, which shed light on the crucial role of cues to action in promoting positive health behaviors. Compared to individuals with low perceived cues to action, those with high cues to action were more likely to engage in all four HBP subscales (i.e., physical activity, stress management, nutrition, and interpersonal connections) as well as the overall health-promoting behaviors. This finding was further strengthened by a Polish study that found that cues to action improve health beliefs and knowledge, which in turn influence behaviors that promote health (60), and a study conducted in Indonesia that found a statistically significant relationship between cues to action and hypertension prevention behavior among adults (61). The observed differences health-promoting behaviors between those with high and low cues to action highlight how crucial outside influences and circumstances are in influencing people's decisions and actions regarding their health. High cues to action are indicators to prioritize one's health and wellbeing that function as prompts, motivators, or reminders. These indications can originate from a variety of places, including medical professionals, friends, family, the media, local initiatives, and personal experiences. The results also suggest that improving cues to action through interventions may be an effective approach for encouraging people to adopt healthy habits. Healthcare providers and public health professionals can support individuals in recognizing and responding to cues that promote positive health actions by raising individuals' awareness of the significance of health behaviors, offering pertinent information and resources, and establishing supportive environments that facilitate behavior change.

There is a statistically significant difference in the overall HPB and in each of its subscales, with the exception of interpersonal relations. Participants with a high-risk perception of NCDs were more likely to engage in overall health-promoting behavior as well as the subscales measuring spiritual growth and stress management. These results were consistent with research done among adults in Diepsloot township, South Africa (27). The result could be explained by the fact that individuals with high perceptions of non-communicable diseases (NCDs) may engage in health-promoting behaviors like stress management and spiritual growth to cope with anxiety and worry. Spiritual growth provides meaning and direction, inspiring healthy habits. By incorporating spiritual growth and stress management, individuals adopt a holistic approach to their overall wellbeing, realizing that all aspects of health are interrelated. Participating in spiritual growth activities can provide a supportive community, increasing adherence and sustainability over time.

### Strength and limitation of the study

The greatly appreciated aspect of the study was the use of the Health Belief Model, which is used to understand how a person's beliefs about specific health behaviors affect such behaviors. The understanding of some of the components that influence the health-enhancing practices led to the development of special interventions. Also, the findings validated the ability of data-driven decision-making as the capability of the policymakers implementing specific intervention measures that will help to reduce the prevalence of NCDs within the Gedeo Zone. As for the study's limitations, one could find it more challenging to ascertain the causal-effect relationship due to its cross-sectional approach. It's possible that the self-reporting questionnaire utilized in this study contributed to a recall bias in the study variables. We employed the mean score to calculate the constructs of health belief models and health-promoting behavior (total and individual constructs) using Likert scales. However, computing a mean presupposes equal intervals, which may not adequately reflect the underlying data. Other limitations of the study were that it had a sample of 705; this may not have included adequate variation of the Gedeo zone's population; it had issues with selection bias; and, may not have captured adequate representation to enable comparison and identification of populations especially at greater risk for NCDs. The study recruitment might present some biases and the proposed health belief model might fail to consider social, economic or other environmental factors that may affect the health behaviors of people. The model may also not capture the cultural stream of health beliefs and their temporal stability which may not be well captured in cross-sectional research designs. Such limitations can limit the applicability and validity of the findings in other public health intervention research. To gain a deeper understanding of the health promotion behaviors of the community in the study area, further research using mixed (qualitative and quantitative) methods and longitudinal designs is required.

## Conclusion

In the study, the mean score of health-promoting behaviors was low. According to the mean score for the six subscales of health-promoting behavior, spiritual growth was most frequently practiced, followed by the nutrition subscale. The health-promoting behavior that was least frequently performed was physical activity. The variables such as gender, occupational status, educational status, marital status, family history of NCDs, health insurance status, perceived health status, knowledge about NCD risk factors, risk perception of NCDs, expected outcome, cues to action, and self-efficacy showed a statistically significant difference with overall health-promoting behavior. Based on stepwise linear regression analysis, gender, marital status, family history of NCDs, good perceived health status, good knowledge of NCD risk factors, high perceived threat, high expected outcome, high self-efficacy, and high cues to action positively affect health-promoting behaviors. Age, primary school educational status, government employees, merchants, and others in occupational status, moderate household wealth index, and rich household wealth index were negatively associated with overall health-promoting behavior, therefore, we urge policymakers and healthcare planners to create focused campaigns for health promotion that address the unique requirements of various marital status and gender subgroups. For instance, providing gender-specific health education and support programs; additionally, family-based interventions should be considered for those with a family history of noncommunicable diseases (NCDs) to increase knowledge of the influence of genetics on NCD risk and promote healthy lifestyle choices within families. To help people keep a good perception of their health and identify any possible problems early, encourage routine health check-ups and screenings. To increase public understanding of NCD risk factors and health-promoting behaviors, extend community-based education initiatives. Provide behavior change interventions that emphasize raising people's awareness of the dangers associated with non-communicable diseases (NCDs), their conviction that healthy behaviors will lead to beneficial consequences, and their self-efficacy in their capacity to initiate and maintain healthy changes. Establish nurturing surroundings that offer indicators for people to participate in activities that promote health, including community projects or workplace wellness programs. Moreover, implement strategies that are tailored to the challenges encountered by particular populations, such as offering services specifically to older adults, those with lower educational attainment, or households with lower incomes and occupations.

## Data Availability

The original contributions presented in the study are included in the article/supplementary material, further inquiries can be directed to the corresponding author.
