# Corrigendum: Health-promoting behavior and its determinants towards non-communicable diseases among adult residents of the Gedeo zone, South Ethiopia: the application of the health belief model

**DOI:** 10.3389/fpubh.2025.1553920

**Published:** 2025-04-03

**Authors:** Habtamu Endashaw Hareru, Tizalegn Tesfaye Mamo, Mesfin Abebe, Berhanu Gidisa Debela

**Affiliations:** ^1^School of Public Health, College of Health Sciences and Medicine, Dilla University, Dilla, Ethiopia; ^2^Department of Midwifery, College of Health Sciences and Medicine, Dilla University, Dilla, Ethiopia

**Keywords:** Health-promoting Lifestyle Profile II, non-communicable diseases, health belief models, determinants, Ethiopia, health-promoting behavior

In the published article, the reference for [21] was incorrectly cited as [Lim EJ, Noh JH, Kim EY. A Study of factors affecting health-promoting behaviors to young-elderly adults in urban and rural communities. *Int J Bio-Sci Bio-Technol*. (2015) 7:367–74. doi: 10.14257/ijbsbt.2015.7.5.36]. It should be/changed in to [Meemee R, Ohn Khin Khin N, Htike Aung M: Factors affecting health-promoting lifestyles among community residents at East Gyogone Ward, Insein Township. 2021], which is reference 36 in the original article.

In the published article, there was an error regarding the date.

A correction has been made to **[Materials and methods]**, [*Study area, period, and design*], [paragraph one]. This sentence previously stated:

“The research was carried out from November 2023 to January 2024”

The corrected sentence appears below:

“The research was carried out from November 01, 2023 to January 01, 2024”

In the published article, there was an error, regarding a mistake in sample size computation, formula, and reference change.

A correction has been made to **[Materials and methods]**, *[Sample size determination, sampling techniques, and procedures]*, [paragraph one]. This sentence previously stated:

“For estimating a single mean, the sample size was determined using a single population equation [*n* = (Z_(α/2) ^*^ σ^2^)/d^2^] by considering a 95% confidence interval (two-sided, α/2), a margin of error (d) of 3%, and the overall mean score of health promotion lifestyle Profile II (HPLP-II) (mean ± standard deviation (σ) (130.26 ± 22.58)) was obtained from a similar previous study conducted among rural adults in Korea [21], with the consideration of the design effect of three (3) and a 10% non-response rate. Finally, the optimum sample size that was used for the current study was 718. A multi-stage sampling technique was employed to choose the study participants. In the first phase, a lottery method (simple random sampling technique) was used to randomly select two Woreda (Wonago and Bule) and one administrative town (Dilla town) out of a total of eight Woreda and four administrative towns, respectively. Nine kebeles (three from each Woreda and three from the administrative town) were chosen at random in the second phase. In the third phase, the number of households in each of the chosen kebeles was documented, and the total sample size of 716 was proportionally allocated to each kebele.”

The corrected sentence appears below:

“For estimating the sample size, a single mean equation was used $\left(n=\;\frac{{\left(Z_{\alpha /2}\right)}^{2*}\sigma^{2}}{d^{2}}\right)$ by considering 97% CI (Z_α/2_ = ± 2.17), a margin of error (d) of 3%, overall mean score of health promotion lifestyle Profile II (HPLP-II) (mean ± standard deviation (σ) (126.67 ± 21.29) was obtained from a previous similar study conducted among rural adults in Myanmar [36], with the consideration of the design effect of three (3) and a 10% non-response rate. Finally, the optimum sample size that was used for the current study was 782. A multi-stage sampling technique was employed to choose the study participants. In the first phase, a lottery method (simple random sampling technique) was used to randomly select two Woreda (Wonago and Bule) and one administrative town (Dilla town) out of a total of eight Woreda and four administrative towns, respectively. Nine kebeles (three from each Woreda and three from the administrative town) were chosen at random in the second phase. In the third phase, the number of households in each of the chosen kebeles was documented, and the total sample size (782) was proportionally allocated to each kebele.”

In the published article, there was an error on the data.

A correction has been made to [**Materials and methods]**, [*Data collection techniques, procedures, and quality control*], [paragraphone]. This sentence previously stated:

“The period of data collection was from November 1, 2023, to December 1, 2023.”

The corrected sentence appears below:

“The period of data collection was from November 01, 2023, to January 01, 2024.”

In the published article, there was an error in the sample size and response rate.

A correction has been made to **[Result]**, [*Socio-demographic and economic characteristics of the participants*], [paragraph one]. This sentence previously stated:

“Out of the total 718 sample sizes, 705 participants were chosen for this study, resulting in a 98.19% response rate.”

The corrected sentence appears below:

“Out of the total 782 sample sizes, 705 participants were chosen for this study, resulting in a 90.15% response rate.”

In the published article, there was an error for a number.

A correction has been made to **[Result]**, [*Health-promoting behavior of the participants towards NCDs*], [paragraph one]. This sentence previously stated:

“In this study, the overall mean score for health-promoting behavior was 73.88 ± 16.79. The mean scores of the six HPLP subscales were higher for the spiritual responsibility subscale (19.8 ± 9), while the physical activity subscale had the lowest score (6.16 ± 4.37) (Table 3).”

The corrected sentence appears below:

“In this study, the overall mean score for health-promoting behavior was 73.88 ± 16.79. The mean scores of the six HPLP subscales were higher for the spiritual growth subscale (16.8 ± 2.9), while the physical activity subscale had the lowest score (6.16 ± 4.37) (Table 3).”

In the published article, there was an error regarding the headings in Table 2.

The corrected Table 2 and its caption appears below.

**Table 2:** The perception of NCDs and knowledge about NCD risk factors among residents of the Gedeo zone, South Ethiopia region, 2023 (n = 705).

**Variables**	**Frequency**	**Percentage (%)**
**Risk perception of NCDs**		
Low perception	322	45.67
High perception	383	54.33
**Expected outcome**		
Negative	183	26.00
Positive	522	74.00
**Cues to action**		
Low	323	45.80
High	382	54.20
**Self-efficacy**		
Low	312	44.26
High	393	55.74
**Knowledge about NCDs**		
Poor	167	23.69
Good	538	76.31

In the published article, there was an error regarding the Mean (± SD) for Spiritual growth value in Table 3.

The corrected Table 3 and its caption appears below.

**Table 3:** The overall score of health-promoting behavior and its subscales among adult residents of Gedeo Zone, South Ethiopia region, 2023 (n = 705).

**Health-promoting behavior scales**	**Possible range score**	**Mean (±SD)**	**Range**
Health responsibility	9–36	11.6 ± 5.5	2–22
Spiritual growth	9–36	16.8 ± 2.9	9–23
Interpersonal relationship	9–36	13.5 ± 2.8	7–23
Nutrition	9–36	13.6 ± 3.4	5–25
Physical activity	8–32	6.16 ± 4.4	0–22
Stress management	8–32	12.1 ± 2.9	4–19
Overall score of HPLP II	52–208	73.88 ± 16.8	38–115

In the published article, there was an error word B to β.

A correction has been made to **[Result]**, *[Factors associated with health-promoting behaviors*], [Paragraph one]. This sentence previously stated:

“A separate regression analysis was used to examine the predictive power of each predictor on the overall HPB score and its subscales. According to the results of the stepwise regression analysis, self-efficacy was the most potent factor impacting behaviors that promote health (B = 14.67, p < 0.01).”

The corrected sentence appears below:

“A separate regression analysis was used to examine the predictive power of each predictor on the overall HPB score and its subscales. According to the results of the stepwise regression analysis, self-efficacy was the most potent factor impacting behaviors that promote health (β = 14.67, p < 0.01).”

In the published article, there was an error regarding the name of ethical approval in the ethical statements.

A correction has also been made to the **Ethics statement**. This sentence previously stated:

“The studies involving humans were approved by Institutional Review Board (IRB) of Dilla University, College of Health Sciences and Medicine. The studies were conducted in accordance with the local legislation and institutional requirements. The participants provided their written informed consent to participate in this study.”

The correct Ethics statement appears below:

“The studies involving humans were approved by Institutional Review Board (IRB) of Dilla University, College of Health Sciences and Medicine, with the ethical protocol number duchm/IRB/044/2023. The studies were conducted in accordance with the local legislation and institutional requirements. The participants provided their written informed consent to participate in this study.”

In the published article, there was an error in the text. The β = was missing and p needed to be in italics.

A correction has been made to **[Result]**, [*Factors associated with health-promoting behaviors*], [paragraph one]. This sentence previously stated:

“cues to action (5.16, p < 0.01).”

The corrected sentence appears below:

“cues to action (β = 5.16, *p* < 0.01).”

In the published article, there was an error in the text. The number 2 needed to be superscript.

A correction has been made to **[Result]**, [*Factors associated with health-promoting behaviors*], [paragraph three]. This sentence previously stated:

“This model showed that 55.7% of the variation in stress management could be explained by all significant factors (p < 0.01, R2 = 0.557, adj. R2 = 0.549)”

The corrected sentence appears below:

“This model showed that 55.7% of the variation in stress management could be explained by all significant factors (*p* < 0.01, R^2^ = 0.557, adj. R^2^ = 0.549)”

The authors apologize for this error and state that this does not change the scientific conclusions of the article in any way. The original article has been updated.

